# Drugs as Chemical Weapons: Past and Perspectives

**DOI:** 10.3390/toxics11010052

**Published:** 2023-01-04

**Authors:** Vladimír Pitschmann, Zdeněk Hon

**Affiliations:** 1 Faculty of Biomedical Engineering, Czech Technical University in Prague, Sítná sq. 3105, 272 01 Kladno, Czech Republic; 2 ORITEST spol. s r.o., Čerčanská 640/30, 140 00 Prague, Czech Republic

**Keywords:** chemical weapons, chemical warfare agents, medicaments, pharmacology, medicine, riot control agents, non-lethal chemical weapons

## Abstract

The emergence of modern chemical weapons and chemical warfare is traditionally associated with World War I, but the use of poisons in the military has its roots deep in the past. The sources of these poisons have always been natural agents that also served as medicines. This relationship between poison and medicine, and nowadays between chemical warfare and medicine, or between ‘military chemistry’ and pharmacy, appears to be very important for understanding not only the history but also the possible future of both phenomena. This article looks at some historical examples of the use of drugs as chemical weapons and, conversely, the use of chemical weapons as medicines. It seeks to find answers to some questions that are particularly relevant to the implementation of the Chemical Weapons Convention, which aims to achieve a world without chemical warfare.

## 1. Introduction

The existing state in the field of chemical weapons is determined, along with the associated dramatic reduction of chemical arsenals, by the Chemical Weapons Convention. The report of the Conference of the States Parties to the OPCW (Organization for the Prohibition of Chemical Weapons) of 1 December 2021 shows that 70,548.6 tonnes of chemical weapons (toxic chemical agents) had been destroyed worldwide by the end of 2020, which is 97.6% of the declared stock. Of these, Category 1 chemical weapons (toxic chemicals) made up 98.3%, i.e., 69,300 tonnes; 278 tonnes of sarin, 115 tonnes of VX and 797 tonnes of sulphur mustard (yperite), totalling 1190 tonnes, were not destroyed by that date [1]. Research and production on new chemical warfare agents (CWA) have virtually stopped or are being conducted secretly. While the military significance of chemical weapons has diminished, their psychological effect persists, as observed in the context of the phenomenon of terrorism. Yet there is a quite open talk of a new generation of what is referred to as non-lethal chemical weapons or of various alternatives to contemporary riot control agents (RCA). Both of these weapon categories (not to be confused!) are, on the face of it, ‘progressive’ in that they can reduce the brutality of armed conflict or increase the effectiveness of crime fighting. On the other hand, their toxicity and, consequently, the safety of their use are problematic in some situations. For RCAs, massive misuse of anti-human repression is an ethically problematic matter.

Chemical weapons can be viewed from a political, military, ethical, legal, or purely scientific/technological perspective; the last-mentioned aspect is going to prevail in this paper. In the classical view, sources of chemical weapons involve natural poisons and toxic industrial agents or their structural analogues—often, the latter covers chemical compounds as a by-product of chemical, biochemical, or pharmaceutical research and development. The role of pharmacy (and medicine as such) in the development of chemical weapons in the past and in thinking about their future seems to be of particular interest. This text takes a closer look at the relationship between a drug and a chemical weapon in order to highlight some of the known and lesser-known links, offering some futurological insights. Using selected examples, it shows that the link between a drug and a chemical weapon (resp. between a chemical weapon and a drug) has a very long history and internal logic; understanding it allows us to find a method to better grasp some modern trends that are related to chemical weapons in one way or another. 

The authors consider it necessary to emphasise that the present text is not a guide to any illegal use of drugs as chemical weapons; indeed, it has been written with a sincere effort to draw attention to some of the pitfalls of scientific and technological development and to enhance awareness of the crucial importance of the ethics of scientific work as such. Its conception follows the previous work by the authors, which discussed the current state and perspectives of chemical weapons [2] and the military significance of toxins [3]. 

## 2. Traditional Agents

### 2.1. Arrow Poisons

Even prehistoric people coated arrowheads with natural poisons when hunting wild animals or in war. The studies of traditional hunting techniques of indigenous tribes and ethnic groups in various parts of the globe provide some insight. During evolution, arrow poisons have often been modified (seemingly simplified), but each time they contained active agents or admixtures that were also used to treat various diseases (Table 1). Similarly to the rhetorical question about the chicken and the egg, the question is what came first: poison as a medicine or poison as a weapon? 

A textbook example is *curare*, the arrow poison of the indigenous peoples of South America, which, however, does not have a uniform composition, containing extracts from various plants of the Menispermaceae family (*Abuta*, *Chondrodendron* and *Curarea* genera) and of the Loganiaceae family, including the *Strychnos* genus, a member of what was formerly the Strychnaceae family. The species of Menispermaceae, used by the natives to treat abdominal pain, colic, eye inflammation and snake bites, relieve fever, stimulate menstruation, treat dropsy and insanity, as well as fungal and other skin diseases [11], are of particular interest in folk medicine. The founders of modern pharmacy in the 19th century tested *curare* as a treatment for malaria or epileptic seizures; later on, its active agents were used in anaesthesiology for surgical procedures requiring muscle relaxation—a field previously dominated by opium [12]. Typical neuromuscular blocking agents (myorelaxants) found in *curare* include the alkaloids tubocurarine and C-toxiferine. A new generation of myorelaxants is represented by synthetic curaremimetics such as succinylcholine (structurally close to the neurotransmitter acetylcholine). Succinylcholine is distinguished by its rapid onset and intensity of action, short block time and quality; it is rapidly degraded in the body by the action of pseudocholinesterase. A certain connection between modern medicine and military art is therefore obvious. The research on succinylcholine (suxamethonium) in the 1950s was closely related to what is referred to as *Tammelin esters* [13], the de facto prototypes in the research on the nerve CWA of series V. In addition, succinylcholine was one of the first fillings for rifle cartridges to immobilise large animals. There is now discussion involving the possible misuse of curaremimetics as “non-lethal chemical weapons” (more on these is covered below).

South American natives have also developed arrow poisons from frog secretions (e.g., the *Phyllobates* genus) containing the extremely toxic batrachotoxin, a sodium channel blocker. Studies of this steroid alkaloid and its analogues point to possible applications in the treatment of heart disease and as anaesthetics. Toad (*Bufo*) secretions containing steroidal cardiotonics or the hallucinogenic alkaloid bufotenine (or methoxy-derivative), which is structurally similar to the neurotransmitter serotonin, may also be a source of arrow poison [14,15]. In antiquity and the Middle Ages, toad poisons were used as a poisoning agent; there is anecdotal evidence that in the 17th century, they were designed to prepare poisoned artillery shells. In folk medicine, they were used in the treatment of dropsy, inflammatory diseases or as an analgesic [16], and recent pharmaceutical research on the poisons is focused on the treatment of cancer and heart disease [17].

The “Big Three” African arrow poisons consist of plant extracts from numerous species of *Strophanthus*, *Acokanthera schimperi* (both in the Apocynaceae family), and *Parquetina nigrescens* from the Periplocacea family. They contain ouabain (g-strophanthin), convallatoxin and other cardioactive glycosides that increase the contractility of the heart muscle and slow down the heart rhythm and the transmission of nerve signals [5]. Convallatoxin and the cardioactive glycosides known as antiarin are also contained in the extract of *Antiaris toxicaria* from the Moraceae family, one of the main constituents of Asian arrow poisons. Strophantins and other cardioactive glycosides have traditionally been used in the treatment of heart failure and as antiarrhythmic agents. Their use in the prevention or treatment of cancer has also recently been studied [18,19]. However, the mechanism of the cytotoxic effect of these agents is not well-understood. One of the new directions of research is the study of the possibilities of improving the anticancer properties of the agents by appropriate modification of the stereochemistry of the molecules [20].

In Eurasia, arrow poisons made from the extract of plants of the genus *Aconitum* (family Ranunculaceae) containing aconitine and related toxic alkaloids, which act on the nerves controlling the heart rhythm and on the heart muscle, prevailed, in addition to the species *Antiaris toxicaria* mentioned above. In India and China, such extracts (such as the “king of drugs” in Tibet) have also been used in medicine since ancient times, e.g., in the treatment of bronchial disorders, hypotension or diabetes, to relieve toothache, as a diuretic or chemotherapeutic agent in the treatment of cancer, etc. [21]. In medieval China during the Song Dynasty (10th to 13th century), chemical hand grenades, which were launched with large slings, were designed to be filled with primary gunpowder (with a rather low nitrate content) mixed with *Aconitum* or other poisonous plants [22]. It cannot go unnoticed that Taoist alchemists used gunpowder alone as a medicine as well.

The ancient Gauls used arrow poison from the species *Taxus baccata* (Taxaceae family), also known as an ingredient in witches’ ointments. The poison contains nitrogenous compounds called taxanes that cause death by respiratory paralysis and cardiac arrest. The cytotoxic effect of taxanes and their analogues is used by modern medicine in the chemotherapy of breast and prostate cancer. Examples include paclitaxel (taxol), a drug extracted from *Taxus brevifolia* and semi-synthesised from baccatin III (*Taxus baccata*), or docetaxel, a drug in the form of a semi-synthetic derivative of 10-deacetylbaccatin III (*Taxus baccata*) [23,24].

### 2.2. Other Natural Poisons (Toxins) Notable from the Military Aspect 

Some protein and non-protein toxins were the subjects of quite intensive chemical resp. toxin weapons research in the 20th century. Botulinum toxin and ricin are textbook examples of protein toxins simultaneously known as a drug and a weapon. This relationship can also be demonstrated with the non-protein saxitoxin, a group of dangerous marine poisons studied in pharmaceutical and military laboratories [7]. It is certainly no coincidence that ricin and saxitoxin are on the list of chemicals included in the Annex on Chemicals (Schedule 1) to the Chemical Weapons Convention [25]. 

Botulinum toxin, produced by the bacterium *Clostridium botulinum*, blocks the release of the neurotransmitter acetylcholine from nerve endings; of a group of seven antigenically distinct proteins, botulinum toxin A is the most potent known poison. The achieved level of biotechnology enabled the Allies to develop botulinum toxin aerial bombs and shells as early as World War II. During the “Cold War”, botulinum toxin projectiles for special rifle weapons were considered. In medicine today, this bacterial poison has found use in the treatment of a wide range of disorders such as chronic migraine, some forms of dystonia, blepharospasm, haemifacial spasm, spasticity, hyperhidrosis, sialorrhea, strabismus, achalasia and other conditions [26].

Ricin is a protein toxin of plant origin, an extremely potent cell poison from *Ricinus communis*. Its effects were already known in prehistoric times and, in ancient times, was described as an ingredient of toxic chemical mixtures, as can be seen, e.g., from the Indian treatise entitled *Arthashastra,* which probably originated from the turn of the 4th and 3rd centuries BC [27]. During World War I, two concepts of chemical ricin munitions were designed based on both poisonings by contaminated shrapnel and the release of a toxic aerosol, neither of which was standardised. Military interest in ricin grew during World War II when field tests on the effectiveness of converting ricin to a combat state in aerosol form were conducted. Its introduction into ordnance was limited by its low stability and challenges in industrial production [28]. Today, ricin is considered a potential agent of terrorism. However, it is also of interest to biomedical research and clinical medicine. It seems that with the help of advanced biotechnology, the cytotoxic effects of ricin could be harnessed to treat cancer [29]. In addition, protein engineering methods can be used to combine the catalytic domain of ricin with the binding domain of interleukin-2 as an immune system protein to create a hybrid toxin that more selectively kills cancer cells [30]. Studies of ricin transport into cancer cells using nanocarriers may be beneficial [29]. The medical use of ricin has so far been hampered by its extreme toxicity. Structurally close to ricin is the toxin abrin from the species *Abrus precatorius*, whose leaves and seeds were used by ancient Indians as an aphrodisiac, laxative or expectorant but were also of military interest (see, e.g., *Arthashastra*). This dual view of this toxin is also evident today [31]. 

Saxitoxins, produced by Dinoflagellata, marine organisms, have recently appeared in chemical weaponry, taking the form of poisoned projectiles or similar special weapons. Experts believe that they can also be used to induce cases of mass inhalation poisoning, although the aerosol is not optimally stable. Tests on mice in military laboratories have shown that inhalation administration can be up to 10 times more effective than intravenous administration [7]. The estimated lethal inhalation concentration for humans is 5 mg·min/m^3^, i.e., 15 times lower than sarin (GB) [7]. Other non-protein neurotoxins of sea origin, produced by algae, cyanobacteria and bacteria, have similar military potential, examples of which include tetrodotoxin, palytoxin, maitotoxin and α-conotoxin that can, like saxitoxin, cause inhalation and percutaneous poisoning. Modern medical research on saxitoxins and their analogues has shown that they can be long-term anaesthetics in the treatment of anal fissures and chronic headaches [32,33]. Tetrodotoxin may also be of therapeutic value in the treatment of pain [34]; palytoxin and related agents may be useful in the prevention and treatment of leukaemia [35]. Research into the therapeutic potential of these substances is not yet complete.

### 2.3. Agents Affecting the Psyche

The era of modern chemical weapons in the 20th century brought about a new concept of drugs as a weapon, which is also confirmed by the experience of research, development and standardisation of so-called incapacitating agents in the first decades of the Cold War. The initial concept of these weapons was mainly based on the study of natural psychoactive agents, which were of key cultic and medical importance to various tribes and ethnic groups across continents, but also served military purposes by greatly increasing the body’s resistance to mental and physical stress under extreme circumstances such as war. However, some synthetic analogues have proven to be even more effective.

In the 1930s, Sandoz Pharmaceuticals, a Swiss pharmaceutical company based in Basel, studied ergot alkaloids (the source of *Claviceps purpurea*) as potential circulatory stimulants. During this research (1938), Albert Hofmann synthesised a number of new agents based on ergobasine (ergoline) or lysergic acid. The best known of these is lysergic acid diethylamide (lysergide), known as LSD-25, for which Hofmann discovered (1943) a remarkable hallucinogenic effect on himself [36], about 4000 times greater than that of mescaline, the strongest known psychedelic agents up to that time, sourced from the cactus *Lophophora williamsii*. Shortly after World War II, LSD-25 was proposed for the treatment of patients suffering from schizophrenia [37], and in the 1950s to 1960s, the US Chemical Corps tested LSD-25 (EA 1729) as a chemical weapon. LSD-25 in aerosol form demonstrated exceptional efficacy (incapacitating concentration ICt_50_ = 30 to 55 mg·min/m^3^), but apparently, high production costs prevented its introduction into ordnance. 

In 1926, Parke-Davis, a Detroit-based pharmaceutical company, synthesised phencyclidine [1-(1-phenylcyclohexyl) piperidine, PCP] and marketed it as Sernyl in the 1950s. This prototype of dissociative anaesthetics, N-methyl-d-aspartic acid (NMDA) receptor blockers, had unique properties, but some side effects limited its use in human medicine; however, it tested well as an immobilising agent in wildlife trapping. Phencyclidine aerosol produces an anaesthetic effect at a concentration of 25 to 50 mg·min/m^3^ and an incapacitating effect (eliminating activity or disabling fighting) at a concentration of 1000 mg·min/m^3^; its lethal p.o. dose is about 100 mg, and death occurs through cardiac and respiratory failure. The US Chemical Corps, which studied phencyclidine as an incapacitating agent EA 2148, introduced it into the ordnance around 1960 under the code name SN. However, standard ammunition was never produced [38]. In the past, the use of phencyclidine dart guns used to immobilise animals has been considered for the purpose of maintaining public order and security [39].

In 1952, Hoffmann-La Roche, the pharmaceutical company from Basel, synthesised 3-quinuclidinyl benzilate (QNB), initially a promising spasmolytic for the treatment of gastric disorders and ulcers [40,41,42]. The high anticholinergic activity of QNB, detected in comparative tests with atropine in rabbits, intrigued the US Chemical Corps enough to study it under the code EA 2277 or BZ. The incapacitating effect (ICt_50_) for inhalation of BZ aerosol is about 170 mg·min/m^3^, and the lethal effect (LCt_50_) is about 200,000 mg·min/m^3^ (about 30 times more effective than SN). Several hundred structurally related glycolates were synthesised during the course of the research, including agents with an oily consistency that can induce percutaneous poisoning [43,44]. Due to its high efficacy and relatively simple production, BZ was introduced into the ordnance (around 1961) and produced in quantity totalling 50 tonnes for aerial cluster bombs. Structurally similar substances with anticholinergic action (atropine, benactyzine) are known antidotes in AChE inhibitors poisoning [45].

### 2.4. Lethal CWAs 

The backbone of modern chemical weapons are lethal CWAs. Their genesis and possible perspectives are also related to pharmacology. For illustrative purposes, arsenic compounds will be given as examples, but in particular blistering agents such as mustard gas and nerve agents will be listed (Table 2).

Inorganic arsenic compounds (e.g., auripigment and realgar) were already used as medicines by the ancient Greeks, Romans and Chinese. This tradition of treating various diseases and cancers has persisted into modern times [47], and, for example, arsenic is being studied as a potential chemotherapeutic agent in the treatment of leukaemia [48]. A new era of medical use of arsenic compounds was opened at the beginning of the 20th century by Paul Ehrlich with the synthesis of the drug Salvarsan, an organic arsenamine with significant antisyphilitic activity. While arsenic compounds were the most widely used murderous poison in the past (e.g., the “poison of the Borgias” in the 15th century or *Aqua Tofana* in the 17th century), it is less known that in the pre-industrial era, in medieval China, and later in Europe and the Arab countries, the compounds were also used in warfare, e.g., in gunpowder shells. A few centuries later, during and after World War I, organic arsenic compounds became widespread as irritants (diphenylchlorarsine, diphenylcyanarsine, 10-chloro-5,10-dihydrophenarsazine) but also as lethal agents such as the blistering ethyldichloroarsine, methyl dichloroarsine or 2-chlorovinyldichloroarsine (lewisite). During World War II, 2,3-dithiopropanol (dimercaprol) was developed as an antidote to poisoning by lewisite (British anti-lewisite, BAL) and is still used today in various modifications to treat acute arsenic, mercury or lead poisoning [49].

The main member of the blistering CWA is sulphur mustard or yperite, bis-(2-chloroethyl)sulphide, first prepared in 1822 by César Despretz. It was introduced into the ordnance during World War I, in which it proved to be a persistent CWA with unusual percutaneous action, and immediately after its first deployment (July 1917), changed the existing character of chemical warfare; later its nitrogen analogues methyl-bis(2-chloroethyl)amine, ethyl-bis(2-chloroethyl)amine and tris(2-chloroethyl)amine were also introduced into the ordnance. However, blistering agents are not known to have ever been used for medical purposes or the subject of pharmaceutical research. Although the clinical signs of poisoning were known, the mechanism of toxic action remained unclear. It is only since about the early 1940s that we have known that they are highly reactive alkylating agents with cytostatic effects and that their mechanism of action involves binding to DNA and cross-linking, which inhibits DNA replication and cell growth. This finding was the birth of a new era of cancer therapy [50,51]. While it was effective in leukaemia, sulphur mustard was not used in clinical practice because of its high toxicity. Instead, nitrogen mustard-based anticancer agents (e.g., chlormethine, chlorambucil, cyclophosphamide) have emerged, which have a long history of clinical use but are also limited by adverse reactions and low selectivity. It seems that a promising strategy in further research on anticancer agents may cover the investigation of nitrogen mustards based on hybrid molecules with introduced “druggable” fragments such as brefeldin A, evodiamine, oridonin, etoposide and tyrosine [52]. 

CWA are the highlight of chemical weapons development to this date, with their action based on inhibiting acetylcholinesterase (AChE). Organophosphorus compounds, which were developed as a by-product of insecticide research, are the main members of these agents referred to as nerve agents. Initially, in the 1930s, a group around Gerhard Schrader synthesised compounds in Germany now known as G-type agents (tabun GA, sarin GB), then during the war, Richard Kuhn and his colleagues synthesised the related soman (GD) while studying vitamins and in the 1950s work in Sweden, the UK and Germany opened the way to V-type agents (now VX, RVX, CVX). The therapeutic potential of nerve agents seems to be irrelevant, although they have left a noticeable mark on medicine, stimulating and increasing interest in the detailed study of the cholinergic transmission of nerve impulses, which has led to important findings in physiology and clinical biochemistry. Investments in research on nerve agents also had an impact on the development of pesticides, optimising their use in agriculture and controlling their health effects. Let us mention that research is currently underway, e.g., on the treatment of Alzheimer’s disease, which is closely related to cholinergic transmission disorders. The administration of cholinesterase inhibitors (e.g., donepezil, galantamine, rivastigmine) increases the amount of synaptic acetylcholine and has been shown to delay the progression of symptoms [53].

### 2.5. Irritants

Since ancient times, irritating fumes prepared by burning natural materials have been used in wars, and later coal dust, quicklime and other loose materials were sprayed. But even in the age of modern chemical weapons, the role of irritants can be significant. At some stage in World War I (at the beginning of trench warfare), the use of irritants was even the trigger mechanism for the deployment of what were systemically lethal agents such as chlorine, phosgene, diphosgene, hydrogen cyanide and later sulphur mustard (yperite). The current range of irritants is extremely diverse. In addition to the already mentioned arsenic compounds, capsaicin and its analogues, CS and CR, are particularly noteworthy for their highly effective ability to act on sensory nerve receptors and to induce the typical symptoms of exposure–severe watering and burning of the eyes, as well as irritation of the respiratory tract and the skin [46]. Mostly known as riot control agents (RCAs), they are commonly and extensively used by police and security forces to maintain order at the intra-national level (Table 3).

The first of these is capsaicin, an alkaloid contained in the *Capsicum* plant species, which is, among other things, a traditional component of arrow poisons (to speed up the absorption of the poison into the blood) and vernacular remedies (toothache and other odontological problems or hastening difficult births) [11]. During World War I, capsaicin, mixed with other chemicals, was experimentally designed for hand and artillery shells. It was later standardised as an RCA along with its synthetic derivatives (morpholide or pelargonic acid vanillylamide). However, it appears that capsaicinoids may also have modern medical applications in the treatment of chronic (neuropathic) pain [55], possibly arthritis, musculoskeletal pain, postoperative nausea and vomiting; they additionally cause apoptosis (programmed death) of prostate cancer cells [56]. In addition, some studies confirm that medically undesirable skin irritation is reduced by the application of compounds in the form of polymeric nanocapsules [57].

In the 1950s, the British security forces introduced the irritant CS (o-chlorobenzylidene malononitrile) as a replacement for chloroacetophenone (CN). When the U.S. military used some formulations of CS on a large scale (about 7000 tonnes) in the Vietnam War in combat against guerrillas, the results were often fatal when applied in high concentrations or confined facilities (underground tunnels) [58]. Currently, CS is the most widely used RCA agent. In 2020, the latest available data, 121 Chemical Weapons Convention countries reported using CS from a total of 138 countries declaring the use of RCAs; the second most used RCA is chloroacetophenone [1], although it is five times less effective. Therapeutic applications of CS are unlikely, but some other benzylidene malonitrile derivatives may be beneficial because of their antibacterial activity [59] or may be used to treat acute lymphoblastic leukaemia [60]. 

During the 1960s and 1970s, CS was supplemented with CR, i.e., dibenzo[b,f]-1,4-oxazepine, which is 5 to 10 times more potent, has a faster onset of action, a higher safety index and, due to its better solubility, is more suitable for operational use in liquid form (e.g., water cannon). Structural analogues of CR, e.g., dibenzoxazepine (Loxapine), may be effective in the prevention and treatment of circulatory diseases, especially angina pectoris and arrhythmia, or in antipsychotic medication (treatment of schizophrenia) [61]. Some dibenzoxazepines may facilitate cholesterol breakdown [62].

## 3. A Shortlist of Other Agents

### 3.1. Synthetic Opioids

The use of opium, the juice of the immature *Papaver somniferum* fruit containing morphine and related alkaloids, has had a long tradition in medicine and the military (pain relief or psychic stimulation). Modern pharmaceutical and organic synthesis methods now offer a variety of structurally different agents, referred to as synthetic opioids, which have much stronger effects due to their higher affinity for opioid receptors (metopon, dextromorphan, etorphine, etc.).

Fentanyl, prepared in the early 1960s by the Belgium-based Janssen Pharmaceuticals, is a notable synthetic opioid along with its numerous analogues, e.g., sufentanil, remifentanil and carfentanil. Fentanyl has a 200–300 times greater anaesthetic effect than morphine, and carfentanil is even more potent: 4000–6000 times [63], and even up to 10,000 times, according to some sources (Table 4). In human medicine, fentanyl injections are administered as a very strong analgesic in anaesthesiology for neuroleptanalgesic anaesthesia in high-risk operations. Also notable is the inhalation effect of synthetic opioids. Administering fentanyl as an aerosol is particularly beneficial in patients whose medical condition (e.g., polytrauma) does not allow intravenous administration. The inhaled therapeutic dose that produces an analgesic effect in humans (0.1 to 0.3 mg) is comparable to the intravenous dose [64]. In addition, synthetic opioids can enter the body percutaneously; for example, fentanyl can be medically administered via a transdermal patch. In general, these are fast-acting analgesics, which act at a higher rate than morphine, but for a shorter period of time. They have a very low therapeutic index and a number of undesirable side effects such as respiratory depression; the most effective agents of this type, such as carfentanil, have no application in human medicine for these reasons and are used, for example, to restrain large animals (including in aerosol form). Naloxone, an opioid receptor antagonist, is used as an antidote.

The interest in fentanyl and its analogues as chemical weapons can be illustrated by two examples. First, in the early 1990s, they were studied in the context of the U.S. program to develop incapacitating weapons, specifically the chemical grenade for riot control [66]. Secondly, their extraordinary effectiveness was demonstrated in October 2002 during an anti-terrorist intervention by Russian special forces against terrorists in Moscow’s Dubrovka Theatre. After an attack with an aerosol of synthetic opioids (apparently a mixture of carfentanil and remifentanil) [67], all the people in the theatre fell asleep, but more than 10% of them died from the effects of the poisoning. 

Despite their extreme toxicity, these agents are widespread in the drug abuse scene as they produce narcotic effects at much lower doses than morphine-based alkaloids. They are also available on illegal online narcotic drug marketplaces called “darknet markets”. Some countries, especially the USA, have long been faced with waves of deaths following overdoses of fentanyl or its analogues [68].

### 3.2. Peptide Bioregulators

One often-discussed problem that can be used to demonstrate the drug–chemical–weapon relationship is that of peptide bioregulators, natural organic compounds (short chains of amino acids) that act as neurotransmitters and hormones and, even at extremely low concentrations, regulate numerous activities in the human body such as blood pressure, heart activity, respiration, muscle contraction, body temperature, sleep, emotions and immune function. It turns out that peptides shorten the process of protein synthesis so that they can slow down the ageing process. Of particular medical importance are short polypeptides, which penetrate more easily through the walls of the intestines and skin into the bloodstream, are safer to administer (they have a higher therapeutic index and fewer side effects) and, unlike conventional medicines, can be recycled by the body. They can be used, e.g., to treat hypertension, prevent thrombosis, strengthen the immune system, kill pathogenic microorganisms, etc. All this may also have military significance. Until recently, polypeptide bioregulators were obtained in minute quantities from natural sources. However, modern biotechnology allows production on an industrial scale so that the compounds can not only be widely used in medicine but can be laboured into ammunition and used as chemical weapons. Examples of such peptides are cholecystokinin, neurokinin or endothelin.

### 3.3. Modern Methods of Research and Development

The well-known CWAs are the result of traditional methods of scientific research and development. While these traditional methods may lead to further improvements in CWAs (e.g., unusual structural analogues, compound formulations or binary chemical munitions components), science and engineering now have much more effective tools at their disposal. In particular, the methods that are successfully used in medicine and pharmaceuticals may, in the future, even break boundaries that were formerly taboo. For example, combinatorial chemistry, protein engineering, brain research and nanotechnology make it possible to find and prepare toxic compounds (often at the interface between chemistry and biology) with programmed effects that can revolutionise the knowledge about drugs and treatments while changing our ideas about chemical weapons and chemical warfare as such. Most of these modern technologies are suitable for the synthesis and application of compounds with molecular weights higher than traditional CWAs. However, this handicap can be compensated to some extent by more advanced methods of conversion to a combat state and propagation (dispersion) in the target area. In principle, however, it is assumed that the CWA domain may primarily be in the field of RCA (or resp., non-lethal chemical weapons), for which a low molecular weight and gaseous or liquid state are not even expected.

## 4. Considerations on the Possible Use of Certain Agents

### 4.1. Use of Certain Agents as Lethal Chemical Weapons

Some groups of drugs are of interest to military and security experts, as a number of studies (including the OPCW) have pointed out or the anti-terrorist crackdown in Moscow suggests. This knowledge and experience, therefore, leads us to consider certain groups of drugs, such as, for example, those modelled on synthetic opioids, as militarily usable lethal chemical weapons, i.e., weapons capable of inflicting high irreversible medical losses on an adversary. Only CWAs can constitute the measure of the effectiveness of such weapons as a kind of comparative standard in that they present the peak of chemical weapons development to date; this clearly involves nerve agents, whose mechanism of toxic action is based on AChE inhibition. Why are these CWAs considered the most dangerous? Due to their extreme toxicity and advantageous physical and chemical properties, they can cause both serious inhalation poisoning (vapours of G-type agents, e.g., GB, GD, GF) and dangerous percutaneous poisoning (V-type agents, e.g., VX, RVX, CVX, probably also Novichok group agents). Research into the military uses of natural poisons has confirmed that although many bacterial toxins are far more toxic than nerve agents (e.g., botulinum toxin is about 100 times more potent than VX when inhaled as an aerosol), their combat effectiveness in the field is lower (the area affected by botulinum toxin is about three times smaller than that of VX) [7]. Moreover, toxins are more challenging in terms of production technology and are not very stable in the field, even under normal climatic conditions. Consequently, the only real threat is posed by inhalation poisoning from aerosol toxins; the stability is, however, questionable in this case. For the possible use of certain drugs, there is an analogous problem. If we consider synthetic opioids, then, for example, the lethal toxicity of carfentanil is close to that of some nerve agents (G-type), but it is much more difficult to produce a lethal concentration of its aerosol in open terrain over a large surface area. As with toxins, the percutaneous efficacy of synthetic opioids is controversial, and the agents are expensive to produce, so they are unlikely to replace nerve agents. 

### 4.2. Use of Certain Agents as RCA/Non-Lethal Chemical Weapons

This necessarily brings us back to the consideration of drugs as non-lethal chemical weapons. Let us note that the safety index (the ratio of lethal to effective concentration) is virtually meaningless when evaluating nerve agents, as their IC_t_ and LC_t_ values when inhaled are very close (for GB, the safety index is about 1.4). However, the safety index of, for example, synthetic opioids is a key parameter that indicates the possibility of their military/police use in a wide range of aerosol concentrations in the field, i.e., from concentrations causing a threshold effect through an incapacitating effect to lethal poisoning. State-of-the-art methods of aerosol generation in field conditions (e.g., smoke grenades, aerosol generators) allow for achieving a concentration of about 100 mg/m^3^, which approximately corresponds to an incapacitating concentration of the psychoactive agent BZ. Achieving such a concentration of carfentanil aerosol would very likely lead to the immobilisation of the affected persons.

Taking into account the efficacy limit (ICt_50_) and safety index, it is clear that synthetic opioids as potential RCA/non-lethal chemical weapons are no match for commonly used irritants. In a certain tactical situation, their advantage may be the unusual nature of the effect (typical of the Moscow case—falling asleep, knocking out the attackers, which would not be realistic with the use of irritants). However, unlike irritants, the effect of synthetic opioids is extremely long-lasting (up to several tens of hours), so their use as classical RCAs, e.g., to suppress unauthorised demonstrations, is practically excluded.

The key question of whether the drugs can be used as RCAs or non-lethal chemical weapons has become particularly urgent following the deployment of fentanyl analogues in the anti-terrorist operation by Russian security forces, the largest of its kind. Discussions and disputes are taking place at several levels:

1. Was the use of these agents legal within the meaning of the Chemical Weapons Convention?

2. Is the designation of any physiologically active group of chemicals as a “non-lethal chemical weapon” justified?

3. Is the concept of RCA sufficiently understandable, or is it problematic and opens up new possibilities for the research and development of chemical weapons?

4. Where are the limits to the development of RCAs/non-lethal chemical weapons?

5. To what extent does all this relativise the meaning of the Chemical Weapons Convention?

Since these are fundamental issues and key questions of contemporary theory and practice in the field of security and defence, we will try to answer them at least briefly.

1. The use of synthetic opioids was legal, but only in the sense that its purpose was to incapacitate the perpetrators of a highly criminal act (terrorism), not to seriously injure or kill hostages, and that it was not used as a means of warfare, i.e., in war (this is prohibited by the Chemical Weapons Convention). 

2. Regardless of the planned and actually achieved objectives of the operation (disabling terrorists), a significant number of the affected persons died as a result of synthetic opioid poisoning. This experience provides significant support for the view that the term ‘non-lethal chemical weapons’ is inaccurate (‘less lethal chemical weapons’ is more correct) and should therefore be excluded from the technical discussion or at least strictly defined. The high mortality rate at Dubrovka is incompatible with the purposes of using the RCA. One of the main counter-arguments is that synthetic opioids appear to have been used without previous operational experience, in an unusual situation and under unusual conditions, de facto in a closed facility, i.e., not in an open space where the use of RCA is usually envisaged. 

3–4. The Chemical Weapons Convention does not define any term ‘non-lethal chemical weapons’. It only defines an RCA as “any non-listed chemical that is capable of producing rapid sensory irritation or overwhelming physical effects in humans that disappear within a short period of time after exposure has ceased”. However, synthetic fentanyl-based opioids and other classes of drugs do not meet this definition of RCA. On the contrary, much closer to this definition are the irritants CS, CR and capsaicin, as well as their synthetic analogues commonly used in police and security practice. However, a significant number of experts also have serious reservations about irritants. Irritant effects are usually limited to mild and transient inflammation of the eyes and skin, but many cases of severe complications and even death are known [54]. There have also been repeated calls for a moratorium on the use of irritants in order to study their long-term effects in detail [69]. However, it seems that this research is still poorly supported and has not yet produced the expected results. On the other hand, the possibilities for finding new irritants are virtually unlimited, the limits perhaps only being set by the laws of nature. All standardised irritants/RCAs are surpassed in their potency by, e.g., the relatively recently discovered resiniferatoxin (RTX), contained in *Euphorbia* latex, which reaches a value of 16 billion SHU on the Scoville scale, i.e., 1000 times higher than capsaicin. RTX can be an effective chemical weapon, but it also has considerable therapeutic potential in analgesia [70].

5. The Chemical Weapons Convention is aimed at the military use of chemical weapons and at eliminating or limiting the possibility of waging chemical warfare on any scale or in any form. The fact that the Convention tolerates the use of chemical toxicants for law enforcement purposes, including domestic riot control, has historical context and justification. Another question is whether and to what extent these agents are abused for purposes not permitted by the Convention. However, the legal research and use of these agents (RCA) in the sense of the Convention and the continuous upgrading of carriers, ammunition and weapons systems maintain the level of chemical armament at such a level that in the event of a war conflict, there is a real threat of deployment of chemical weapons (chemical warfare). The essence of this mechanism can be illustrated by the experience of World War I, when the initial use of ammunition filled exclusively with irritants/tear-producing agents (e.g., ethyl bromoacetate, chloroacetone, bromoacetone, xylyl bromide or *o-dianisidine*) seemingly ‘legalised’ the subsequent massive use of chemical agents based on lethal agents, first chlorine near Ypres in April 1915.

### 4.3. Additional Notes on the Role of Medicine and Pharmacy

We have already mentioned the antidotes (i.e., drugs) used in CWA poisonings in several places. It is certain that the role of medicine and pharmacy in the field of prevention and protection against chemical weapons is diverse and irreplaceable. As for antidotes, we have to admit that they are only available against some types of CWA so far, although they include the most important ones (nerve agents); a brief overview of the possibilities of antidote therapy is presented in Table 5. However, the set of potentially abusive poisons is so extensive that the issue of antidotes and the possibilities of their practical use appears to be a difficult problem to manage. Paradoxically, similar modern methods and procedures can be used in their development and applications as in the development of CWA (e.g., combinatorial chemistry, aerosol applications, microencapsulation, nanotechnology, etc.).

Other remarks relate to the very use of drugs as chemical weapons. It is clear that their use for these purposes would violate the Geneva Protocol (1925), the Chemical Weapons Convention (1993) and the Biological and Toxin Weapons Convention (1972). The use of drugs as chemical weapons creates the risk of seriously undermining the trust in medicine as an eminently humane field and may confront doctors and medical staff with serious ethical issues. If drugs are used for these purposes, their source will be the pharmaceutical industry. On the one hand, controlling the use of drugs (especially highly toxic or affecting cognitive functions and the immune system) is important from the point of view of the proliferation of chemical weapons, but on the other hand, controlling the research, development and production of drugs should not limit the freedom of scientific research and the solution of urgent medical tasks.

## 5. Conclusions

The presented text on the misuse of a drug as a chemical weapon and the beneficial use of chemical weapons as a medicine leaves ample room for reflection and further discussion, although it primarily deals with the classical conception of these phenomena. The latest scientific findings, which are used in various areas of biotechnology (genetic engineering, synthetic biology, etc.), are, with a few exceptions, left aside for the time being. However, several key conclusions can even be drawn from the classical concept:

(a) For virtually all groups of known CWA and militarily significant toxicants, a direct or indirect link to drugs or medical applications can be found. This is due to the fact that the basic principle of both categories is the same—the physiological effect.

(b) In the past, the use of a drug as a chemical weapon (or CWA) was rather incidental, as a by-product of pharmaceutical research and the circumstances. Recently, however, modern methods have made it possible to plan and programme this research to a large extent.

(c) The discovery and introduction of nerve agents (organophosphorus inhibitors of AChE) of the G and V series marked the peak in the development of CWAs as a principal component of chemical weapons; it seems that in this respect, the discovery of the so-called ‘latest generation CWAs’ based on Novichok-type agents did not bring any apparent progress.

(d) Opportunities for further development may be seen in research into non-lethal or less lethal chemical weapons in the field of both RCAs and possible military use. The object of this research may involve, for example, calmatives, psychoactive agents, peptides and other physiologically active agents synthesised and studied at top pharmaceutical institutes.

(e) The role of the Chemical Weapons Convention remains crucial, although its enforcement depends on the current military–political situation in the world.

## Figures and Tables

**Table 1:** Characteristics of selected arrow poisons and toxins.

Compound	Source	Effect	LD_50_, μg/kg	M_r_ (Relative Molecular Mass)
Tubocurarine	*Chondrodendron* spp.	Myorelaxant	130 (mouse, i.v.) [4]	609.7
Ouabain	*Strophanthus* spp.	Cardioactive glycoside	110 (cat, i.v.) [5]	584.7
Antiarin	*Antiaris toxicaria*	Cardioactive glycoside	120 (mouse, i.v.) [6]	566.6
Aconitine	*Aconitum* spp.	Interaction with Na-channels	110 (rat, i.v.) [7]	645.7
Botulinum toxin	*Clostridium botulinum*	Acetylcholine blockade	0.001 (mouse, i.v.) [8]	150 000
Ricin	*Ricinus communis*	Inhibition of protein synthesis	3 (mouse, i.p.) [9]	62 000
Saxitoxin (STX)	Dinoflagellata	Blockade of Na-channels	10 (mouse, i.p.) [10]	299.1

**Table 2:** Characteristics of some deadly CWA [46].

CWA	M_r_ (Relative Molecular Mass)	LCt_50_ Inhal. mg·min/m^3^	LD_50_ p.c. (Liquid) mg·min/m^3^	LCt_50_ p.c. (Vapour) mg·min/m^3^
Sulphur mustard/yperite (HD)	159.1	1000	1400	10,000
Nitrogen mustard (HN-3)	204.5	1000	1400	10,000
Lewisite (L, L-1)	207.3	1000	1400	5000
Tabun (GA)	162.1	70	1500	15,000
Sarin (GB)	140.1	35	1700	12,000
Soman (GD)	182.2	35	350	3000
VX	267.4	15	5	150
RVX (R-33)	267.4	15 (?)	5 (?)	150 (?)

HD: bis(2-chloroethyl)sulphide; HN-3: tris(2-chloroethyl)amine; L: dichloro(2-chlorovinyl)arsane; GA: ethyl-(dimethylphosphoramido)cyanidate; GB: isopropyl-methylphosphonofluoridate; GD: (3,3-dimethylbutan-2-yl)-methylphosphonofluoridate; VX: S-[(2-diisopropylamino)ethyl]-O-ethyl-methylphosphonothiolate; S-[(2-diethylamino)ethyl]-O-isobutyl-methylphosphonothiolate.

**Table 3:** Characteristics of irritants (RCA) [54].

Agent	Onset of Effect, Seconds	Duration of Effect, Minutes	Relative Efficacy	ICt_50_ mg·min/m^3^	LCt_50_ mg·min/m^3^
CN	3–10	10–20	1	20–50	8500–25,000
CS	10–60	10–30	5	4–20	25,000–100,000
CR	Instant	15–60	20–50	0.2–1	>100,000
OC	Fast	30–60	Not known	Not known	>100,000

**Table 4:** Relative efficacy, therapeutic index and lethal dose for selected synthetic opioids (according to different sources) [65].

Compound	Relative Efficacy	Therapeutic Index	LD_50_ (Rat, i.v.), mg/kg
Morphine (standard)	1	70	<200
Methadone	4	12	Not known
Alfentanil	75	1100	47.5
Remifentanil	220	33,000	Not known
Fentanyl	300	300	3.5
Sufentanil	4500	25,000	17.9
Carfentanil	Up to 10,000	10,600	3.4

**Table 5:** Overview of antidotal therapy options for poisoning with selected CWAs (listed in the Annex on Chemicals of the Chemical Weapons Convention).

CWA	Mechanism of Action of CWA	Antidote Type	Example, Note
Nerve agents	AChE inhibition	Functional	Atropine
		Causal, AChE reactivator	Oximes (HI-6, pralidoxime)
Sulphur mustard	Cytostatic agent, alkylating agent	Not available	Supportive drugs only (sodium thiosulfate)
Lewisite	Alkylating agent, arsenic effect	Thiol groups	BAL (dimercaprol)
Hydrogen cyanide	Blockage of cellular respiration	Methemoglobin formation	Sodium nitrite
		Conversion to thiocynates	Sodium thiosulfate
		Formation of cyano-complexes	Hydroxocobalamin
Phosgene	Destruction of the pulmonary blood-brain barrier	Not available	Supportive drugs only
BZ	Anticholinergic effect	AChE inhibition	Physostigmine
Ricin	Inhibition of protein synthesis	Development in progress	Antitoxin (biotechnology) [71]
Saxitoxin	Sodium-ion channel blocking	Development in progress	4-aminopyridine tested on animals [72]

## Data Availability

Not applicable.
